# Redefining the expressed prototype *SICAvar *gene involved in *Plasmodium knowlesi *antigenic variation

**DOI:** 10.1186/1475-2875-8-181

**Published:** 2009-07-31

**Authors:** Stacey A Lapp, Cindy C Korir, Mary R Galinski

**Affiliations:** 1 Emory Vaccine Center, Yerkes National Primate Research Center, Emory University, 954 Gatewood Rd, Atlanta, Georgia, USA; 2 Division of Infectious Diseases, Department of Medicine, School of Medicine, Emory University, Atlanta, Georgia, USA

## Abstract

**Background:**

The *SICAvar *gene family, expressed at the surface of infected erythrocytes, is critical for antigenic variation in *Plasmodium knowlesi*. When this family was discovered, a prototypic *SICAvar *gene was characterized and defined by a 10-exon structure. The predicted 205-kDa protein lacked a convincing signal peptide, but included a series of variable cysteine-rich modules, a transmembrane domain encoded by the penultimate exon, and a cytoplasmic domain encoded by the final highly conserved exon. The *205 SICAvar *gene and its family with up to 108 possible family members, was identified prior to the sequencing of the *P. knowlesi *genome. However, in the published *P. knowlesi *database this gene remains disjointed in five fragments. This study addresses a number of structural and functional questions that are critical for understanding *SICAvar *gene expression.

**Methods:**

Database mining, bioinformatics, and traditional genomic and post-genomic experimental methods including proteomic technologies are used here to confirm the genomic context and expressed structure of the prototype *205 SICAvar *gene.

Results

This study reveals that the *205 SICAvar *gene reported previously to have a 10-exon expressed gene structure has, in fact, 12 exons, with an unusually large and repeat-laden intron separating two newly defined upstream exons and the *bona fide *5'UTR from the remainder of the gene sequence. The initial exon encodes a PEXEL motif, which may function to localize the SICA protein in the infected erythrocyte membrane. This newly defined start of the 205 *SICAvar *sequence is positioned on chromosome 5, over 340 kb upstream from the rest of the telomerically positioned *SICAvar *gene sequence in the published genome assembly. This study, however, verifies the continuity of these sequences, a 9.5 kb transcript, and provides evidence that the 205 *SICAvar *gene is located centrally on chromosome 5.

**Conclusion:**

The prototype *205 SICAvar *gene has been redefined to have a 12-exon structure. These data are important because they 1) address questions raised in the *P. knowlesi *genome database regarding *SICAvar *gene fragments, numbers and structures, 2) show that this prototype gene encodes a PEXEL motif, 3) emphasize the need for further refinement of the *P. knowlesi *genome data, and 4) retrospectively, provide evidence for recombination within centrally located *SICAvar *sequences.

## Background

The phenomenon of antigenic variation in malaria parasites was first described in rhesus macaques infected with *Plasmodium knowlesi *[1]. SICA (Schizont Infected Cell Agglutination) variant antigens were identified in *in vivo*-derived clonal parasite lines as large parasite-encoded proteins (between 180–220 kDa), and were confirmed to be expressed at the surface of trophozoite and schizont-infected erythrocytes [2]. The 190 kDa and 210 kDa SICA antigens expressed in the Pk1(A+) clone were switched *in vivo *to the alternative expression of 200 kDa and 205 kDa SICA antigens that define the resulting Pk1(B+)1+ clone, as previously described [2]. In 1999, the *P. knowlesi SICAvar *gene family responsible for antigenic variation was reported, and the first *SICAvar *gene, encoding the 205 kDa SICA antigen of clone Pk1(B+)1+ was characterized as having a complex 10-exon structure [3]. This gene structure was surprising because it sharply contrasted with the 2-exon structure reported a few years earlier for *Plasmodium falciparum var *genes [4,5]. Nevertheless, despite the high level of diversity expected of a large family responsible for antigenic variation, proteomic methods have clearly demonstrated a direct relationship between the *P. knowlesi SICAvar *and *P. falciparum var *gene families [6].

The *205 SICAvar *gene, characterized prior to the advent of the *P. knowlesi *sequencing project, was identified by screening Pk1(B+)1+ gene expression libraries with specific antisera, followed by PCR, RT-PCR, 5'RACE and 3'RACE procedures to compile the reported 10-exon gene structure [3]. These investigations also revealed a genomic alteration at the 3' end of the *205 SICAvar *gene compared to the gene in parental Pk1(A+) cloned parasites [3]. Later studies mapped this alteration in greater detail and made the distinction that two alleles existed: the *205A SICAvar *allele in the Pk1(A+) cloned parasites and the *205B SICAvar *allele in the Pk1(B+)1+ cloned parasites [7]. These two *SICAvar *alleles showed apparent identity until the final intron. The *205B SICAvar *allelic phenotype in the Pk1(B+)1+ parasites is the outcome of a recombination event that occurs within the final intron of the *205A SICAvar *gene in Pk1(A+) cloned parasites with the specific placement at this junction of an alternative *SICAvar *intron, cytoplasmic-domain-encoding final exon, and 3'UTR sequence [3,7]. The recently reported *P. knowlesi *genome sequence [8] is based on gDNA of the Pk1(A+) cloned parasite line and, therefore, should contain the sequence of the *205A SICAvar *allele and also provide insights regarding the source of the *SICAvar *DNA sequence that recombined with this gene to generate the *205B SICAvar *allele.

Importantly, Corredor and colleagues [7], working with contigs from an early genome assembly, further showed that the highly conserved final *SICAvar *exon was present in approximately 108 copies in the genome, and that *SICAvar *genes had unusually conserved yet polymorphic 3' UTR sequences. The 3' UTR has expansive repeated motifs with the GGTTTCACACC(T)*n*^1^GTCC(T)*n*^2 ^(*n*^1 ^= 4–8; *n*^2 ^= 21) consensus sequence that includes the GGGTT(T/C)A *Plasmodium *telomeric repeat motif [9,10] immediately after the stop codon, followed by A/T rich homopolymeric segments that differ in size for each *SICAvar *gene family member, and then a small region with more balanced GC content [7], reviewed in [11]. The discovery of the recombination event associated with the *in vivo *switch in the variant type and the presence of these unusual 3' UTR gene structures, suggested that the 3' end and UTR characteristics of *SICAvar *genes may be relevant with regards to the genetic or epigenetic mechanisms that control expression and switching of this gene family [7], reviewed in [11].

Several additional large and diverse gene families involved or implicated in the production of variant antigens and proposed switching mechanisms have also been discovered in *Plasmodium *[12-16]. Transcriptional control and epigenetic mechanisms have been shown to be paramount in the determination of which *P. falciparum var *gene is expressed [17], but the complete repertoire and precise mechanisms that govern gene expression and switching among *var *(or other) gene family members remain elusive. To this end, it is critical to be able to relate *in vitro *findings with what occurs *in vivo*, in the context of the host environment, which is known to influence antigenic variation [11]. In this regard, it is particularly noteworthy that cloned *P. knowlesi *parasites passaged through splenectomized rhesus monkeys cease to express SICA at the surface of the infected red blood cell [18]. Moreover, cloned SICA [-] parasites were shown to be less lethal than SICA [+] clones in rhesus monkeys [18].

The *P. knowlesi *rhesus macaque system remains unchallenged as the best *in vivo *model for studies of antigenic variation in *Plasmodium*. While this model provides direct knowledge into *SICAvar *gene regulation and SICA expression, it can also provide insights regarding similarities and differences in the molecular mechanisms governing *var *gene expression [11]. Moreover, *P. knowlesi *has *kir *genes, which are members of the large *pir *gene family identified in species lacking "*var*" gene orthologs (e.g. *P. vivax *and rodent malaria species) [8,19]. The roles this family may play in antigenic variation or pathogenesis in primate and rodent malaria infections remain uncertain. Importantly, investigations of both *SICAvar *and *kir *gene expression can be undertaken *in vivo *using *P. knowlesi *in rhesus monkeys and not only through *in vitro *culture. Because studies of gene expression, especially for such large complex gene families, require precise knowledge relating to the genes' UTRs, introns/exons and genomic context, it is especially critical that the *P. knowlesi *genome sequencing data is refined. Here multiple approaches to correct, confirm, and redefine the prototypic *205 SICAvar *gene in *P. knowlesi *bring emphasis to this point.

## Methods

### Data mining and bioinformatics

The GeneDB *P. knowlesi *database developed and hosted by the Wellcome Trust Sanger Institute, and the BLAST server at the Sanger Institute website, were used to locate the published *205A SICAvar *gene sequence [3,7] and identify additional genomic sequence related to this gene and other *SICAvar *gene family members. All primers designed for this study were screened by BLAST searches to ensure appropriate specificity (Table 1). A synteny map including the *P. vivax *and *P. falciparum *genomes was generated using the PlasmoDB genome browser [20]. The redefined *SICAvar *gene sequence with 12 exons was manipulated *in silico *to remove the predicted intron sequences, translate the coding sequence, and analyse the newly predicted 205 kDa SICA protein. The redefined *205 SICAvar *gene sequence has been submitted to NCBI, and the original accession numbers AF078128 and AF078130 have been revised.

**Table 1:** Primer List

Primer	Sequence	Amplicon
PKH051981.-1449For	GGAGAAGGAATGTGGATAACGAATG	5'UTR
PKH051981.46Rev	TCTTCTTCATCCATTCTTCCAACAA	

PKH051981.27For	GGAAGAATGGATGAAGAAGACTG	Exon 1-exon2 of PKH_051981
PKH051981.946Rev	ATATCGTCTCTAAGGTCTGCATTAG	
PKH051981.946RevT7	TAATACGACTCACTATAGGGAGGATATCGTCTCTAAGGTCTGCATTAG	
PKH051981.482For	ACATCCATCGAACCACATGAA	
PKH051981.985Rev	GGTGTAACTGCCGTCAGTTAA	

205A/B EXON1.66For	CACTTCAAATCTGACGACACTAATG	Revised exons 2–4 across gap
205A/B EXON1.62For	TGGACACTTCAAATCTGACGAC	
205A/B EXON2.653Rev	GATGTTCCAGGTTTAGTCCCC	
205A/B EXON2.390Rev	GCCACTTCCTCCTATCTTAGCAC	
205A/B EXON1.222Rev	CGCTGCTGCTTCATCTCTTG	

PKH051981.849For	GATTTTTGCGGAGAGGACTG	Non-coding region downstream of exon 2
PKH051981.838For	GAATAGGAATGAGGAAGAAGCC	
PKH051981.526For	GGCTGTCGCATTGAACGAAC	
PKH051981.2874Rev	CCTAAGCAACCTTCTTTCCTTG	
PKH051981.1681Rev	TCCCTTCCATTCCTTCCCTCTC	

PKN385d08.45For	CCTTCTTTCCATCATTCTTTCATCC	Non-coding region upstream of PkH_000630
PKN385d08.260Rev	CGCTGCTGCTTCATCTCTTGTC	

PKH051981.526For	GGCTGTCGCATTGAACGAAC	Non-coding region downstream of PKH_051981
PKH051981.838For	GAATAGGAATGAGGAAGAAGCC	
PKH051981.849For	GATTTTTGCGGAGAGGACTG	
PKH051981.1681Rev	TCCCTTCCATTCCTTCCCTCTC	
PKH051981.2874Rev	CCTAAGCAACCTTCTTTCCTTG	

205exon11gsp7.Rev	GTTCAACTTCTTTCTGCTTATTTCCC	Exon 11 oligoprobe

205exon2.1F	CAGCAATGACGAAGGATGGAATC	Exon 4 probe
205A/B EXON2.653Rev	GATGTTCCAGGTTTAGTCCCC	

### *Plasmodium knowlesi *parasite infections, RNA, gDNA, and infected red blood cell membranes

Pk1(A+) and Pk1(B+)1+ clonal parasites were propagated in rhesus macaques as originally described [2]. Total RNA from synchronous ring-stage parasites was extracted in Trizol LS (Invitrogen), precipitated with 100% ethanol, and purified with RNeasy mini columns. DNase I digest was done on the column for 15 minutes. A260/A280 ratios of 2.0 are typical for RNA prepared by this method. Additionally the quality of RNA samples used in these studies was verified with an Agilent bioanalyzer 2100. Genomic DNA stocks were prepared from mature schizonts and extracted with a midi QIAamp DNA blood kit (Qiagen). Purified infected red blood cell membrane preparations for LC-MS/MS were prepared as described [21], using late trophozoites matured in culture from ring-stage parasites obtained from rhesus macaques.

### PCR, RT-PCR and DNA sequencing

Gene-specific primers (Table 1) were designed for the two *SICAvar *exons contained in PkH_051981, and also to amplify and confirm 1.45 and 1.76 kb of the genomic sequence upstream and downstream, respectively, in both Pk1(A+) and Pk1(B+)1+ parasites. The continuity of the mRNA transcript from PkH_051981 to PkH_000630 was also confirmed with several RT-PCRs and subsequent cloning and sequencing (Additional file 1). For reverse transcription-polymerase chain reaction (RT-PCR) experiments, cDNA was generated with the Thermoscript kit (Invitrogen) by oligo dT-priming 1 ug ring-stage RNA from Pk1(A+) and Pk1(B+)1+ parasites at 57°C for 90 minutes. To test for gDNA contamination, reactions lacking the reverse transcriptase enzyme were included and found to be negative. For PCRs, 5 ng of gDNA [Pk1(A+) or Pk1(B+)1+] was used as a template and amplifications were performed with Platinum Taq Supermix (Invitrogen) for 35 cycles at the appropriate temperatures and extension times. RT-PCR and PCR products were either cloned into the pCR2.1 TOPO-TA vector (Invitrogen) and sequenced with vector primers (M13/T7), or direct sequenced using gene-specific primers. The Big Dye Terminator kit v3.1 and an ABI PRISM^® ^3100 Genetic Analyzer were used to generate sequence data. Clone alignments were generated with MacVector^® ^7.2.2 software.

### Northern blots

Twenty micrograms of Pk1(B+)1+ ring-stage RNA were electrophoresed on a 0.8% LE agarose gel (Ambion) in 1× TBE and transferred to a Hybond-XL membrane (GE Healthcare) overnight with 0.5N NaOH, then UV-fixed with a Stratalinker 1800. A 770 bp RT-PCR gene-specific product of PkH_051981 (from bp 27 of the first exon to near the end of exon 2) was amplified with specific primers, with the reverse primer having a T7 promoter sequence (Table 1). An antisense riboprobe generated from *in vitro *transcribing the PCR product using the T7 Strip-EZ kit (Ambion) was hybridized overnight at 68°C in ULTRAhyb (Ambion) followed by 2 low-stringency washes (2× SSC/0.1% SDS) and 2 high-stringency washes (0.2× SSC/0.1% SDS), each for 30 min at 55°C. Blots were exposed overnight on MS Kodak film at -80°C with an intensifying screen. An exon 11, 26 bp oligonucleotide probe (Figure 2C), based on an antisense gene-specific primer (Table 1), was radiolabeled with the Oligonucleotide 3' End Labeling System (Perkin Elmer), according to the manufacturer's protocols. The oligonucleotide probe was hybridized overnight at 42°C in ULTRAhyb-oligo (Ambion), followed by 2 washes (2× SSC/0.5% SDS) at 42°C.

### Southern blots

One ug aliquots of Pk1(A+)1+ gDNA were individually digested overnight at 37°C with selected restriction enzymes and electrophoresed in a 0.6% agarose gel overnight at 25 volts. The gel was depurinated in 0.25N HCl, denatured in 1.5 M NaCl/0.5 M NaOH, and neutralized with 1.5 M NaCl/1 M Tris/HCl (pH 7.45) before being transferred overnight with 10× SSC onto Hybond-N+ membrane (GE Healthcare). Membranes were UV-fixed (Stratagene Stratalinker 1800) and baked for two hours at 80°C in a vacuum oven.

A 504 bp gene-specific PkH_051981 PCR product from position 482 to 985 (Table 1) was labelled with alpha ^32^P dATP (PrimeIt kit, Stratagene) and annealed overnight at 65°C in hybridization buffer (0.25 M NaH_2_PO_4_/6× SSC/10× Denardt's/56 mM Na_4_P_2_O_7 _*10H_2_O) containing 100 ug/ml salmon sperm DNA, followed by 3 low stringency washes in 2× SSC/0.1% SDS for 30 min at 50°C and 3 high stringency washes in 0.2× SSC/0.1% SDS for 30 min at 55°C. The Southern blots were exposed overnight to several days on MS Kodak film with an intensifying screen at -80°C. After development, blots were stripped with three treatments of boiling 0.1× SDS and re-exposed to confirm the removal of probe. A subsequent hybridization was done with a 330 bp probe representing part of the second exon found on the PkH_000630 contig (for primers, see Table 1) in the same manner as described above.

### Proteolytic digests and liquid chromatography tandem mass spectrometry (LC-MS/MS)

Proteins were analysed by mass spectrometry as previously described [6], with several modifications. Pk1(B+)1+ trophozoite-infected erythrocyte membranes were purified as described [21] and membrane proteins extracted in Laemmli sample buffer and run on a 4–15% SDS-PAGE gradient gel. All protein bands were excised from SDS-PAGE gels and the gel pieces were destained and dried. Dried gel bands were digested at 37°C overnight with 0.4 g of proteomics grade trypsin (Sigma). The resulting peptides were then extracted with 0.1% trifluoroacetic acid in 50% acetonitrile (Sigma), desalted and concentrated using ZipTip pipette tips containing C18 reversed-phase media (Millipore), and then washed in 0.1% trifluoroacetic acid and eluted in 0.1% trifluoroacetic acid/50% acetonitrile (Sigma). Cleaned peptides were analysed at the Emory Proteomics Core Service Center by reverse-phase liquid chromatography coupled with tandem mass spectrometry (LC-MS/MS) using an LTQ-Orbitrap mass spectrometer (Thermo Finnigan). During database searches, a reverse database strategy was used to evaluate false discovery rate; and the matched peptides were filtered according to matching scores to remove all false matches from the reverse database [22]. Only proteins that were matched by at least two peptides were accepted to increase the confidence in the identification.

## Results

### Data mining reveals that a '*SICAvar *fragment' annotated as PkH_051981 in the Pk4 assembly >400 kb distal to the telomere of chromosome 5 is related to the beginning of the 205 *SICAvar *gene

**Figure 1:**
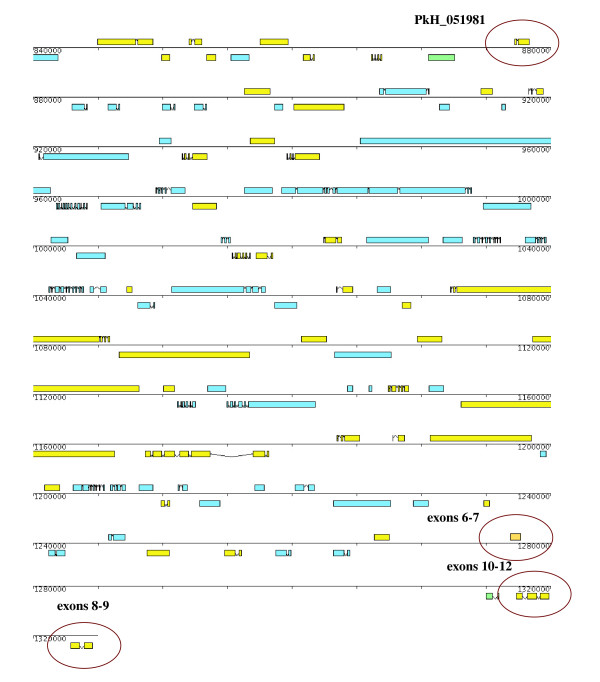
**Genomic context of PkH_051981 and originally defined *205 SICAvar *gene fragments on chromosome 5 in the Pk4 assembly**. The schematic represents *P. knowlesi *chromosome 5 from bp position 840,000 to the end, from Pk4 chromosome maps available from GeneDB. PkH_051981 is centrally located, 340 kb upstream from the other *205 SICAvar *gene fragments positioned at the end of chromosome 5. The telomeric fragments are not found in the correct order, as originally described [3], and they are positioned in this assembly on different DNA strands and orientations. Exons 3–5 are not indicated here, as they are on the unmapped contig PkH_000630. Exons 6–7, 8–9, and 10–12 are on contigs PkH_052810, PkH_052840 and PkH_052830 respectively.

Individual exon sequences from the prototype 10-exon 205 *SICAvar *gene [3,7] were used to BLAST the *P. knowlesi *database at GeneDB (Version 4) to define the location in the genome, and they were found to match a number of '*SICAvar *fragments.' From the outset, this analysis indicated that the organization of the *205A SICAvar *gene was unresolved in the Pk4 assembly [8]. Five *205A SICAvar *gene fragments were identified, assigned to either the plus or minus strands near the end of chromosome 5, or as unmapped contigs not yet assigned to a chromosome (Figure 1 and Table 2). Fragment PkH_051981 containing two *SICAvar *exons was of special interest, given its long distance from the telomeric fragments, 340 kb upstream; exon 1 of this fragment also encodes a PEXEL-like motif with the sequence EEWMK, which may function to transport the expressed SICA protein to the infected red blood cell membrane [23-25].

**Table 2:** *SICAvar *205A allele fragments as annotated in the published *P. knowlesi *genome database, Pk4 assembly [8]

Original Exon	Match in GeneDB	Coordinates on Chromosome 5	Strand Orientation
**1**	PkH_051981, matches genomic sequence upstream of PkH_000630	878189878313, unmapped	plus, minus

**2**	First exon of PkH_000630 'fragment'	unmapped	minus

**3**	Second exon of PkH_000630 'fragment'	unmapped	minus

**4**	Not identified, but located 245 bp upstream of PkH_052810 'fragment'	12766741276757	plus

**5**	PkH_052810	12769191277581	plus

**6**	PkH_052840	13239521324566	minus

**7**	PkH_052840	13228811323579	minus

**8**	PkH_052830	13191921319851	minus

**9**	PkH_052830	13181971318886	minus

**10**	PkH_052830	13173471317778	minus

**Figure 2:**
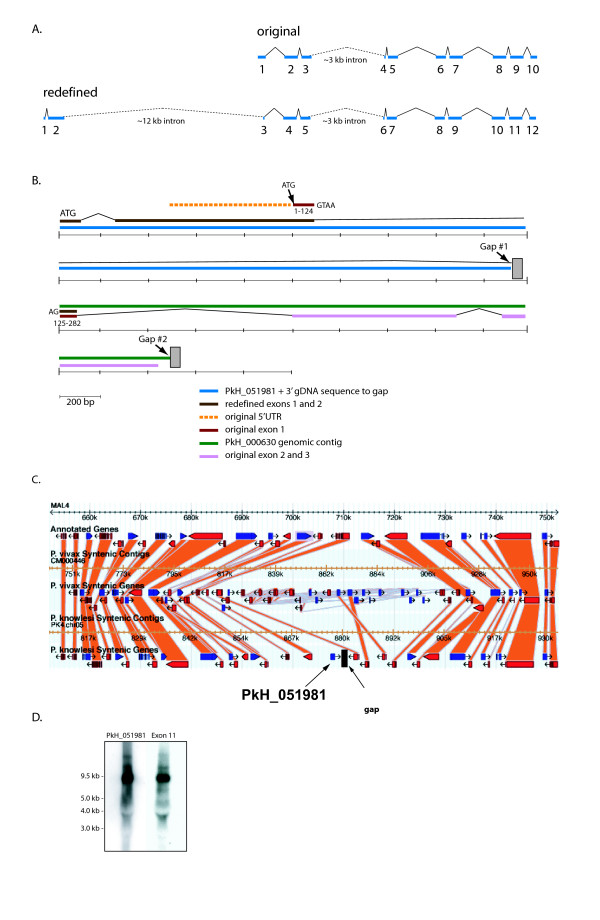
**Redefining the structure of the *205 SICAvar *gene**. A. Schematic representing the genomic organization of the exons and introns of the original (10 exon) and redefined (12 exon) *205 SICAvar *gene. Blue boxes denote exons, black lines denote introns, and dotted lines represent the estimated sizes of uncharacterized 12 kb and 3 kb introns, drawn roughly to scale. B. Schematic representing the discovery of the PkH_051981 two-exon gene fragment and its relationship with the *205 SICAvar *gene. The color-coded bars are drawn to depict the relationship of the newly defined start of the *205 SICAvar *gene with its originally defined 5'UTR and exons 1 through 3. The originally defined 5'UTR and exon 1 sequence are identical to sequence in the PkH_051981 fragment. Part of the originally defined exon 1 was also found to be identical to sequence from the unmapped contig PkH_000630, revealing that the original exon 1 actually contains an internal intron. Previously, this sequence was only available from cDNA generated via 5'RACE experiments and this intron went undetected. See Additional file 1: Alignment of PkH_051981, genomic sequence surrounding PkH_051981, PkH_000630, and sequenced clones. C. Synteny map of the region surrounding PkH_051981 showing many orthologous genes in *P. knowlesi, P. vivax *and *P. falciparum*. PkH_051981 is near the end of a contiguous syntenic block that extends upstream for 70,449 bp. D. Northern blot data supports the premise that the PkH_051981 sequences are part of the *205 SICAvar *transcript. Individual lanes of *P. knowlesi *[Pk1(B+)1+] ring-stage total RNA were hybridized with a PkH_051981 antisense riboprobe and an oligonucleotide probe representing exon 11. The 205 *SICAvar *transcript signal (9.5 kb) is attained with each probe. The standard sizes noted are based on the Millennium RNA markers from Invitrogen.

**Figure 3:**
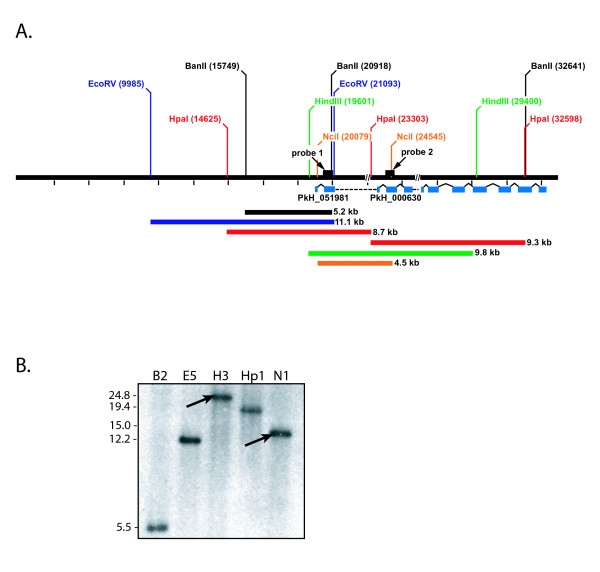
**Southern blot data supports the continuity of PkH_051981 and distal exons of the *205 SICAvar *gene, and the presence of an intron of about 12 kb between exons 2 and 3**. A. Schematic depicting a tentative restriction map covering 35 kb surrounding the *205 SICAvar *locus, based on the premise that the PkH_051981 and distal exons of the *205 SICAvar *gene were contiguous in the genome. The predicted position of the redefined 12-exon *205 SICAvar *gene is noted (blue boxes). The predicted sizes of the various restriction fragments are noted by colour-coded bars, without the size of sequence gaps taken into consideration. The position of the two probes used consecutively in the Southern blot shown below in Figure 3B, are labelled. The presence of two gaps, referring to unresolved sequence in the genome database, are indicated downstream of the position of each probe. B. Southern blot. *P. knowlesi *[Pk1(A+)] gDNA restriction enzyme digests were hybridized consecutively with probes representing the second exon of PkH_051981 (shown) and exon 4 (not shown). Arrows point to fragments that were visualized with both probes, as would be expected, in support of the continuity of these sequences in the genome. The enzymes used were *Ban*II (B2), *Eco*RV (E5), *Hind*III (H3), *Hpa*I (Hp1), and *Nci*I (N1). The expected sizes of *Hind*III, *Hpa*I, and *Nci*I are exceeded by about 13, 9, and 8.5 kb, respectively (Figure 3A and Table 3), supporting the presence of a large intron of close to 12 kb.

Continued bioinformatic investigations led to the development of a redefined model for the *205 SICAvar *gene (Figures 2A and 2B), which is supported by data throughout this report. BLAST searches revealed that 528 bp of the originally reported presumptive 5' UTR *SICAvar *sequence [3,7] and the first 124 bp of the *SICAvar *exon 1 as described in 1999 match the second exon in the PkH_051981 fragment. A typical splice donor sequence (GTAA) marks the end of this second exon, supporting our conclusion that an intron exists within the sequence of the originally reported exon 1. The remaining 158 bp of the original exon 1 (bp 125–282) match part of an open reading frame (ORF) upstream of the 'two exon-containing SICA *var *fragment' encoded in the unmapped PkH_000630 contig (see Table 2). This contig includes the originally defined exons 2 and 3 and introns 1 to 3 of the *205 SICAvar *gene. The splice acceptor preceding the 158 bp exon positioned at the 5'end of PkH_000630 includes a typical long polypyrimidine tract (TTCTTTCATCT_(24)_ACAG). BLAST searches involving sequence downstream of the second exon of PkH_051981 identified other genomic contigs that supplied an additional 406 bp of intron sequence. Combined with the 162 bp of available sequence 5' to the 158 bp *SICAvar *exon in PkH_000630, this intron has 2959 bp of known sequence (Additional file 1). Since there is no overlap between the available genome sequence 3' to PkH_051981 and 5' to PkH_000630, the actual size of the intron positioned between the redefined *SICAvar *exons 2 and 3 (Figures 2A and 2B) was uncertain. However, this intron has been estimated to be at least 12 kb by PCR and Southern blot experiments (see below, Figure 3B). Primers specific to exon 2 of PkH_051981 and corresponding to the ends of the predicted exons 2 and 3 (Figure 2B) of fragment PkH_000630 were tested in PCRs using gDNA from Pk1(A+) and Pk1(B+)1+ parasites. In each case a product over 12 kb was evident but too weak to scan and observe visually. However, this product resisted all attempts at cloning, or direct sequencing, which can be explained due to the presence of extensive repeated motifs in this and comparable regions of other *SICAvar *genes. Lengthy repeated motifs, with one example of 405 bp repeated 2.5 times (Additional file 2), were noted, and the full extent of these repeated sequences remains unknown [26].

Interestingly, BLAST searches of the *P. falciparum *and *P. vivax *genome databases resulted in alignments showing that the PkH_051981 fragment is within a syntenic block (Figure 2C), which corresponds to a relatively central chromosomal location on chromosome 4 in *P. falciparum *(MAL4) and contig CM000446 from *P. vivax *[16,26].

### The continuity of *SICAvar *exons in fragments PkH_051981 and PkH_000630 is confirmed by RT-PCR and sequencing

The bioinformatic analyses discussed above suggested that PkH_051981 contains previously unrecognized upstream sequence of the *205 SICAvar *gene, and RT-PCRs were conducted to evaluate this possibility. Using oligo dT-primed cDNA from both Pk1(A+) and Pk1(B+)1+ ring-stage total RNA, clones were generated and sequenced to confirm the splice sites and predicted mRNA sequence generated from the PkH_051981 sequence (Additional file 1). Most clones reflected spliced intron sequences between the predicted exons 1 and 2 within PkH_051981, but two clones possessed the intron sequence as well. The RT-minus samples were negative, so these two clones could represent pre-mRNA. Multiple RT-PCRs were done to amplify the cDNA sequence from PkH_051981 and PkH_000630. These products were cloned and sequenced and confirm the continuity of the mRNA transcript (Additional file 1).

### PkH_051981 and downstream *205 SICAvar *sequences hybridize to a 9.5 kb transcript on northern blots

To confirm whether the exons within PkH_051981 are in fact part of the 205 *SICAvar *transcript, a gene-specific PkH_051981 antisense riboprobe and an oligonucleotide probe representing the redefined exon 11 sequence (Figure 2A) were tested in northern blot experiments using Pk1(B+)1+ ring-stage RNA. Each probe hybridized to a predominant transcript of about 9.5 kb (Figure 2D). Importantly, the PkH_051981 and the redefined exon 11 (original exon 9, GSP3/GSP7oligonucleotide) sequences hybridize as single-copy sequences on Southern blots, as show in Figure 3B and [3], respectively.

### Southern blot analysis confirms genomic continuity of the redefined *205 SICAvar *gene and indicates that a ~12 kb intron exists between exons 2 and 3

Having developed corroborating evidence through RT-PCR and northern blot experiments that PkH_051981 and the originally reported *205 SICAvar *transcripts are expressed together as one transcript, it was critical to evaluate whether these sequences were contiguous in the genome or separated by 340 kb in the *P. knowlesi *genome database as shown in Figure 1A. As noted above, primers corresponding to the redefined exons 2 and 3 (Figures 2A and 2B) were tested in PCRs and a large band of over 12 kb could be visualized on a gel, supporting the possibility of the genomic continuity of these sequences. To examine this further, a hypothetical restriction map was produced with the presumption that these sequences were contiguous, and also noting the location of two sequence gaps (Figure 3A). This map includes the total available sequence of the *205A SICAvar *allele plus genomic sequence 20 kb upstream of PkH_051981 to the gap downstream of PkH_052830, a span of 34.9 kb. Genomic DNA from Pk1(A+) parasites was digested with a panel of enzymes determined from the predicted restriction map and Southern blots were carried out. A representative Southern blot using five diagnostic enzymes (Table 3; Figure 3B) confirms the general accuracy of the restriction map. The Southern blot was probed first with a gene-specific PCR product representing PkH_051981 (shown) followed by a probe representing exon 4. Common hybridization signals were visible in the *Hind*III and *Nci*I digests supporting the premise that PkH_051981 is in fact contiguous with the rest of the known *205 SICAvar *gene sequence. Thus, it can be concluded that the originally described 10 exons of the *205 SICAvar *gene are located downstream of the PkH_051981 two-exon containing sequence, rather than at the end of chromosome 5, as shown in Figure 1. The specific sizes of the *Hind*III, *Hpa*I and *Nci*I hybridization bands, larger than those predicted in the restriction map based on the absence of sequence gaps (Figure 3A), corroborate the estimated 12 kb size of the intron between exon 2 and 3, as well as the 3 kb intron noted earlier between exons 5 and 6 (Figure 2A) [3]. Despite repeated and persistent efforts in this laboratory, both of these introns were unable to be cloned and sequenced; they also mark the location of sequence gaps in the genome database [8].

**Table 3:** Projected and observed restriction enzymes fragments

Restriction Enzyme Fragment Sizes (kb)				
Probe: PkH_051981				
				
	Projected	Observed	Difference	Spans Sequence Gap(s)
*Ban*II	5.2	Yes	None	No
*Eco*RV	11.1	Yes	None	No
***Hind*III**	**9.8**	**23**	13.2	1 and 2
*Hpa*I	8.7	18	9	1
***Nci*I**	**4.5**	**13**	8.5	1
				
**Probe: Exon 4**				
				
	Projected	Observed	Difference	Spans Sequence Gap(s)

*Ban*II	11.7	22	10	2
*Eco*RV*	>13.6	40+	?	1 and 2
***Hind*III**	**9.8**	**23**	13.2	1 and 2
*Hpa*I	9.3	12	2.7	2
***Nci*I**	**4.5**	**13**	8.5	1

* The gDNA sequence downstream of the *205 SICAvar *gene has not been resolved, thus the location of the next *Eco*RV site is unknown.

### LC-MS/MS identifies the 205 kDa SICA protein in the membrane ghosts of Pk1(B+)1+ infected erythrocytes, with peptide hits to the redefined N-terminus and other previously described regions of this protein

LC-MS/MS was used to confirm that the 205 kDa protein was detected in Pk1(B+)1+ trophozoites, known to express this protein [2,3], and specifically in the infected red blood cell membrane by subjecting the purified infected membrane proteins to LC-MS/MS analysis. In the Pk1(B+)1+ clone, peptides mapping to PkH_051981, PkH_052810, and PkH_052840 were detected from the same pooled protein bands excised from around the 180 kDa to 230 kDa area of an SDS-PAGE gel. PkH_051981 and PkH_052810 were each identified by three unique peptides, which confirms their presence (Additional file 3). In contrast, PkH_052840 was identified by two non-unique peptides suggestive of a shared sequence with other SICA proteins. BLAST analyses of the encoded protein sequences of these fragments identify each of them strictly to the *205 SICAvar *gene with 100% identity.

The redefined *205 SICAvar *gene structure (Figure 2A) was also assessed with regards to its cysteine-rich domains (CRDs). The 205 SICA protein was originally reported to have seven CRDs [3]. The second exon of PkH_051981 encodes six additional cysteine residues, which expands the length of the initial CRD described previously [3]. The redefined 205 SICA protein is comprised of 2,038 amino acids from a deduced ORF of 6,114 bp and has a calculated mass of 227 kDa. An adenosine residue at the -3 position precedes the putative start methionine, which is typical for *Plasmodium *start sites.

### Identification of PkH_000660 as the source of the *205B SICAvar *allele-specific 3' sequences

The availability of the *P. knowlesi *genome sequence, based on sequencing Pk1(A+) gDNA, provides the exciting opportunity to know the original source of the DNA sequences that recombined with the silent *205A SICAvar *allele, *in vivo*, to create the expressed *205B SICAvar *allele [3,7,11]. To determine the origin of this DNA, BLAST searches were performed with the *205B SICAvar *allele-specific sequences. The unmapped contig PkH_000660 matched these sequences with 100% identity. Because this sequence is not mapped in the Pk4 assembly [8], its genomic context remains unknown. However, the fact that this sequence has been reported as unmapped raises the possibility that it may be flanked by typically observed frequent low complexity stretches of A and T nucleotides and/or repeated motifs, which, as also noted above, can be especially challenging to sequence in *Plasmodium*.

## Discussion

In this report, through data mining, bioinformatics and experimental confirmation, the gene structure of the prototype *205 SICAvar *gene has been redefined. The data presented confirms that this gene is comprised of 12 exons *in toto*, instead of 10 as originally reported [3]. The presumptive UTR originally reported for the *205 SICAvar *gene had been in question from the perspective that this sequence hybridized to gDNA digests as a single-copy sequence [7]; BLAST searches also supported this result, indicating that this region contained specific unique sequence. These data contrasted with the nature of 5' UTRs from the *P. falciparum var *genes, since several defined classes of related *var *gene 5' UTRs have been recognized [27,28]. A typical signal sequence was also not clearly recognized in the primary deduced protein sequence reported for the 205 kDa SICA protein [3]; a finding that is consistent with original reports for *var *genes [4,5]. However, PEXEL motifs have since been reported in the erythrocyte membrane proteins (EMP1) encoded by the *var *gene family, with apparent functions related to the transport of these proteins to the infected red blood cell membrane [23-25].

Importantly, the redefined 5'UTR of the *205 SICAvar *gene is in fact a multi-copy sequence that can be classified in one of a few defined groups with regard to potential promoter sequences (unpublished data) and the redefined N-terminal region has a PEXEL-like motif [8] that may supply the required signal sequence for protein translocation of the encoded SICA protein to the membrane of the infected host erythrocyte.

BLAST searches reported here led to the generation of cDNA containing *SICAvar *sequence from PkH_051981 and the upstream part of the originally reported *205 SICAvar *gene. Because the published *P. knowlesi *assembly (Pk4) [8] maps the 5'UTR-containing PkH_051981 fragment on chromosome 5, over 340 kb from the rest of the *205 SICAvar *exons near the end of this chromosome, two possibilities were considered to account for the observed linkage of these exon sequences in cDNAs: 1) that a trans-splicing or co-transcriptional mechanism functioned to unite transcripts produced at the central and distal locations, or 2) that the remaining *205 SICAvar *fragments actually occur immediately downstream of PkH_051981. Together, the DNA and RT-PCR-based data presented here show unequivocally that the centrally located 5'UTR and two-exon-containing PkH_051981 fragment is in fact contiguous with the 10-exon *205 SICAvar *gene sequence. Therefore, the originally reported *205 SICAvar *sequence should be repositioned downstream from the PkH_051981 sequence in the *P. knowlesi *genome database, and the complete 12-exon gene should be annotated as the *205 SICAvar *gene (and, specifically, the *205A SICAvar *allele). The *205 SICAvar *alleles were originally reported on chromosome 4 based on pulse field gel electrophoresis experiments [7], but the *P. knowlesi *genome project has verified this to be chromosome 5 [8].

Northern blot experiments also support the continuity of these sequences, as gene-specific probes representing PkH_051981 and exon 4 both hybridize to the expected 9.5 kb *205 SICAvar *transcript. This report also confirms the size of this *205 SICAvar *transcript, which was previously judged using GTG agarose and formaldehyde gels to be closer to 7.0 kb [3]. Repeated experiments using various probes, updated RNA markers, and LE agarose gels without formaldehyde, show that this transcript is consistently about 9.5 kb.

These data have several important implications. First, our analyses strongly support the view that many of the >200 *SICAvar *gene fragments reported in the *P. knowlesi *genome [8] are, in fact, unassembled segments of *bona fide SICAvar *genes. This is a very different scenario from the alternative view that the genome contains numerous fragments of *SICAvar *genes. Knowing the correct gene family structure is requisite for determining potential gene expression mechanisms. *SICAvar *fragments could reflect pseudogenes or remnants of recombination events, or be modules used by the parasite to help generate diversity, or function in some way to activate or silence specific members of this gene family. If most fragments are actually parts of *bona fide SICAvar *genes, the evolutionary and gene expression considerations would differ. The general organizational picture of the *SICAvar *variant antigen genes could prove to be comparable to the *var *genes in *P. falciparum*, with genes both centrally and telomerically positioned in the genome [27]. The estimate of up to 108 *SICAvar *gene family members [7] provides an upper limit in the *P. knowlesi *genome, while the 29 *SICAvar *genes annotated as full-length in the genome database [8] may reflect only a glimpse into this family and its genomic organization.

It is also worth recognizing that 45 of the 206 fragments (22%) annotated in the Pk4 assembly as *SICAvars *have structures similar to PkH_051981: a short PEXEL-encoding exon and a second exon, which together are termed here as 'PEXEL-containing modules.' If like PkH_051981, other such modules are followed downstream by a very large repeat-laden intron, this would help explain why they, by and large, remain disjointed as annotated fragments followed by gaps in the current genome assembly. In support of this proposition, like PkH_051981, the majority of PEXEL-containing modules (41/45 or 91%) have gaps indicated downstream. Eight of these have more than 2 kb of non-coding sequence reported (between 2.2–6.6 kb) before the start of the gap. These modules, predictably, could be linked to downstream ORFs by unusually large introns. Consistent with this notion, six of the 29 *SICAvar *genes reported in the *P. knowlesi *genome (PkH_072380, PkH_091300, PkH_100210, PkH_110360, PkH_111220, and PkH_132190) have one or more introns between 7.4 kb to 13.6 kb, and all but one of these is found between exons 2 and 3, suggesting that large introns at this position may be a common characteristic of at least a subset of *SICAvar *genes.

As anticipated, when the chromosomal region containing the PkH_051981 fragment was compared with *P. vivax *and *P. falciparum *genome sequences [16,27], synteny was observed for many upstream and downstream genes, giving confidence in the general assembly of the *P. knowlesi *chromosome. *P. vivax *is phylogenetically closely related to *P. knowlesi*, but lacks orthologues of the *SICAvar *(or *P. falciparum var*) gene family [16], and so the absence in *P. vivax *of sequence related to PkH_051981 would be expected.

The newly defined central position of the *205 SICAvar *gene has implications with regards to the nature of the recombination event that occurred within the *205A SICAvar *allele in Pk1(A+) parasites to create the *205B SICAvar *allele in Pk1(B+)1+ parasites. These alleles are distinguished by a recombinant event that occurred within the penultimate intron, resulting in the replacement of an alternative *SICAvar *downstream intron sequence, final exon, and 3'UTR [7]. The current data indicates that this genomic alteration is not simply the result of the increased tendency for recombination of telomeric sequences [29], but the outcome of a recombination event involving centrally located *SICAvar *sequences. Of note, it is also reported here that the alternative *SICAvar *sequence that recombined in the Pk1(A+) parasites to create the *205B *allele in the Pk1(B+)1+ parasites is contained on contig PkH_000660, which in the published Pk4 assembly has not been assigned to a chromosome. Since the *P. knowlesi *genome project was based on sequencing Pk1(A+) gDNA, if the genomic context of this contig were known, it could prove to be especially insightful with regards to the specific genetic mechanism responsible for this recombination event and the associated switch in expression to the 205 kDa SICA protein.

The genomic analysis, sequencing and annotation of members of the large *SICAvar *multigene gene family come with many challenges. Granted, there are as many as 108 *SICAvar *genes, and they have a large complex multi-exon gene structure. However, like the *P. falciparum var *genes, the *SICAvar *genes have many regions that are highly polymorphic as well as regions showing extensive sequence conservation. The introns and UTRs are especially challenging to study due to their lengthy stretches of low complexity sequence comprised of A and T nucleotides or repeated motifs, which can extend for hundreds or even several thousand base pairs. These regions, as exemplified here by the large intron between *SICAvar *exons 2 and 3, can prohibit the thorough and accurate sequencing and assembly of genomic contigs. Therefore, it is not surprising that the *P. knowlesi *database has many gaps associated with these and other difficult regions. The original and continued refinement of the prototype *SICAvar *gene, despite these caveats, provides some optimism that a refined *SICAvar *gene family dataset is within reach.

Further rigorous work is needed at the bench, and smaller-scale, focused sequencing projects directed at specific problem regions could be envisioned. The accuracy of the *SICAvar *gene sequences and genome organization is critical for generating hypothesis-driven studies on the genetic and epigenetic mechanism related to *SICAvar *gene expression and switching of SICA antigens. A more polished genome, ideally reaching closure, is also important for gene expression studies relating to other gene families; for example, those encoding other proteins associated with the infected RBC membrane, such as the KIRS or PHISTS [8,13,16,30] or merozoite proteins like the *P. knowlesi *normocyte binding proteins [31].

## Conclusion

The data reported here, with the redefined *205 SICAvar *gene and encoded protein, is key to the further advancement in our understanding of *SICAvar *gene and protein expression. Studies can now be envisioned to investigate the stage-specific expression and trafficking of this SICA protein (and others) to its position in the red blood cell membrane. An in-depth analysis of promoter regions is also now feasible.

The recent availability of a *P. knowlesi *genome sequence database is a much-welcomed tool, which has proved to be invaluable towards understanding the organization and evolution of *SICAvar *genes in this species. The Sanger Centre can only be commended for the work completed to this point. However, the discrepancy of the available *in silico *data and our experimental confirmation argues for the need of a more advanced genome assembly. While much can and will be done on a small scale in the context of specific research projects, further focused genome-wide sequencing efforts could resolve a majority of the current gaps more quickly. Complete and accurate sequence information is essential for drawing conclusions with regards to genetic or epigenetic mechanisms relating to variant antigen switch events. This is important in light of the fact that *P. knowlesi *is an exceptional *in vivo *and *in vitro *model for investigations of antigenic variation in malaria, and given the specific relationship of the *SICAvar *gene family and the *P. falciparum var *gene family [6,11], this system should be capitalized. Moreover, *P. knowlesi *has been increasingly recognized as the cause of zoonotic infections, illness and death in humans [32,33].

## Competing interests

The authors declare that they have no competing interests.

## Authors' contributions

SAL and MRG made substantial contributions to the conception and design of this research, the analysis of the data, and the drafting of the manuscript. SAL conducted the genetic analyses and all experiments except for the proteomic studies. CCK designed, carried out and interpreted the proteomic experiments. All authors read and approved the final manuscript.

## Supplementary Material

Additional file 1 **Alignment of PkH_051981, genomic sequence surrounding PkH_051981, PkH_000630, and sequenced clones**. A complete alignment of all available genomic sequence from the start of PkH_051981, sequenced clones that span exon 1 to exon 3, and the available genomic sequence of PkH_000630. Click here for file

Additional file 2 **Sequence alignment of the 405 bp perfect tandem repeat identified in intron 2 of the redefined *205 SICAvar *gene**. Analysis using the Tandem Repeats Finder identified a 405 bp perfect repeat in intron 2 of the redefined *205 SICAvar *gene. The alignment of the repeat is depicted. Click here for file

Additional file 3 **LC-MS/MS derived peptide sequences from Pk1(B+)1+-infected RBC membranes that match the predicted protein products of PkH_051981, PkH_052810, and PkH_052840**. A table is presented showing the peptide sequences from Pk1(B+)1+-infected RBC membranes that match the predicted protein products of PkH_051981, PkH_052810, and PkH_052840. Click here for file
