# Supplementary material for: Redefining the expressed prototype SICAvar gene involved in Plasmodium knowlesi antigenic variation
Source: Malar J. 2009 Jul 31;8:181. doi: 10.1186/1475-2875-8-181 (PMC3152765; doi:10.1186/1475-2875-8-181)
Supplement: Additional file 2 — Sequence alignment of the 405 bp perfect tandem repeat identified in intron 2 of the redefined 205 SICAvar gene. Analysis using the Tandem Repeats Finder identified a 405 bp perfect repeat in intron 2 of the redefined 205 SICAvar gene. The alignment of the repeat is depicted. [file 1475-2875-8-181-S2.doc]

Indices: 2889--3902 Score: 2028

Period size: 405 Copynumber: 2.5 Consensus size: 405

2889 AAGACTACTTAGGGGGGGGGGAAAGGGAAGACTACTTAGGGGTAAGGAAAGACTACTTAGGGGTA

1 AAGACTACTTAGGGGGGGGGGAAAGGGAAGACTACTTAGGGGTAAGGAAAGACTACTTAGGGGTA

2954 AGGAAAGACTACATAGGGGTAATGAAGGACTACTTAGGGGTATAGAAAGACTACTTAGGGGTAAG

66 AGGAAAGACTACATAGGGGTAATGAAGGACTACTTAGGGGTATAGAAAGACTACTTAGGGGTAAG

3019 GAAAGACTACTTAGGGCTAAGGAAAGACTACTTAGGGGGAAGGAAGGAAGGTTTAGGAGTAAGGA

131 GAAAGACTACTTAGGGCTAAGGAAAGACTACTTAGGGGGAAGGAAGGAAGGTTTAGGAGTAAGGA

3084 AAGACTACTTAGGGGTAAGGAAAGACTACTTAGGGGTATAGAAAGACTACTTAGGGGTAAGGAAA

196 AAGACTACTTAGGGGTAAGGAAAGACTACTTAGGGGTATAGAAAGACTACTTAGGGGTAAGGAAA

3149 GACTACTTAGGGGTTAGGAAAGACTACTTAGGGGTAAGGAAAGACTACTTAGCGGTAAGGAAAGA

261 GACTACTTAGGGGTTAGGAAAGACTACTTAGGGGTAAGGAAAGACTACTTAGCGGTAAGGAAAGA

3214 CTACTTAGGGGTAAGGAAAGACTACGTAGGGATAAGGAATGAGGGTTTAGGGGTAAGGAAAGACT

326 CTACTTAGGGGTAAGGAAAGACTACGTAGGGATAAGGAATGAGGGTTTAGGGGTAAGGAAAGACT

3279 ACTTAGGGGTATAGA

391 ACTTAGGGGTATAGA

3294 AAGACTACTTAGGGGGGGGGGAAAGGGAAGACTACTTAGGGGTAAGGAAAGACTACTTAGGGGTA

1 AAGACTACTTAGGGGGGGGGGAAAGGGAAGACTACTTAGGGGTAAGGAAAGACTACTTAGGGGTA

3359 AGGAAAGACTACATAGGGGTAATGAAGGACTACTTAGGGGTATAGAAAGACTACTTAGGGGTAAG

66 AGGAAAGACTACATAGGGGTAATGAAGGACTACTTAGGGGTATAGAAAGACTACTTAGGGGTAAG

3424 GAAAGACTACTTAGGGCTAAGGAAAGACTACTTAGGGGGAAGGAAGGAAGGTTTAGGAGTAAGGA

131 GAAAGACTACTTAGGGCTAAGGAAAGACTACTTAGGGGGAAGGAAGGAAGGTTTAGGAGTAAGGA

3489 AAGACTACTTAGGGGTAAGGAAAGACTACTTAGGGGTATAGAAAGACTACTTAGGGGTAAGGAAA

196 AAGACTACTTAGGGGTAAGGAAAGACTACTTAGGGGTATAGAAAGACTACTTAGGGGTAAGGAAA

3554 GACTACTTAGGGGTTAGGAAAGACTACTTAGGGGTAAGGAAAGACTACTTAGCGGTAAGGAAAGA

261 GACTACTTAGGGGTTAGGAAAGACTACTTAGGGGTAAGGAAAGACTACTTAGCGGTAAGGAAAGA

3619 CTACTTAGGGGTAAGGAAAGACTACGTAGGGATAAGGAATGAGGGTTTAGGGGTAAGGAAAGACT

326 CTACTTAGGGGTAAGGAAAGACTACGTAGGGATAAGGAATGAGGGTTTAGGGGTAAGGAAAGACT

3684 ACTTAGGGGTATAGA

391 ACTTAGGGGTATAGA

3699 AAGACTACTTAGGGGGGGGGGAAAGGGAAGACTACTTAGGGGTAAGGAAAGACTACTTAGGGGTA

1 AAGACTACTTAGGGGGGGGGGAAAGGGAAGACTACTTAGGGGTAAGGAAAGACTACTTAGGGGTA

3764 AGGAAAGACTACATAGGGGTAATGAAGGACTACTTAGGGGTATAGAAAGACTACTTAGGGGTAAG

66 AGGAAAGACTACATAGGGGTAATGAAGGACTACTTAGGGGTATAGAAAGACTACTTAGGGGTAAG

3829 GAAAGACTACTTAGGGCTAAGGAAAGACTACTTAGGGGGAAGGAAGGAAGGTTTAGGAGTAAGGA

131 GAAAGACTACTTAGGGCTAAGGAAAGACTACTTAGGGGGAAGGAAGGAAGGTTTAGGAGTAAGGA

3894 AAGACTACT

196 AAGACTACT

Statistics

Matches: 609, Mismatches: 0, Indels: 0

1.00 0.00 0.00

Matches are distributed among these distances:

405 609 1.00

ACGTcount: A:0.37, C:0.09, G:0.35, T:0.19

Consensus pattern (405 bp):

AAGACTACTTAGGGGGGGGGGAAAGGGAAGACTACTTAGGGGTAAGGAAAGACTACTTAGGGGTA

AGGAAAGACTACATAGGGGTAATGAAGGACTACTTAGGGGTATAGAAAGACTACTTAGGGGTAAG

GAAAGACTACTTAGGGCTAAGGAAAGACTACTTAGGGGGAAGGAAGGAAGGTTTAGGAGTAAGGA

AAGACTACTTAGGGGTAAGGAAAGACTACTTAGGGGTATAGAAAGACTACTTAGGGGTAAGGAAA

GACTACTTAGGGGTTAGGAAAGACTACTTAGGGGTAAGGAAAGACTACTTAGCGGTAAGGAAAGA

CTACTTAGGGGTAAGGAAAGACTACGTAGGGATAAGGAATGAGGGTTTAGGGGTAAGGAAAGACT

ACTTAGGGGTATAGA
