# Supplementary material for: Redefining the expressed prototype SICAvar gene involved in Plasmodium knowlesi antigenic variation
Source: Malar J. 2009 Jul 31;8:181. doi: 10.1186/1475-2875-8-181 (PMC3152765; doi:10.1186/1475-2875-8-181)
Supplement: Additional file 3 — LC-MS/MS derived peptide sequences from Pk1(B+)1+-infected RBC membranes that match the predicted protein products of PkH_051981, PkH_052810, and PkH_052840. A table is presented showing the peptide sequences from Pk1(B+)1+-infected RBC membranes that match the predicted protein products of PkH_051981, PkH_052810, and PkH_052840. [file 1475-2875-8-181-S3.pdf]

PKH_051981

K.TAQAAQLVQSAGGGTK.E
K.YLKENADSLASFVK.V
R.YFTAGVGTTTGK.K

PKH_052810

K.VDALAQQAQGSGK.N
R.TACELVAGGLLR.I
K.WIFGGLLDR.M

PKH_114570, PKH_052840, and PKH_040710

K.GPSFEQTMGCLFLK.E
R.KACNHIAAGLK.Y
